# Factors associated with smoking initiation among Saudi male adolescents: A longitudinal study

**DOI:** 10.18332/tpc/109167

**Published:** 2019-06-07

**Authors:** Mutaz Mohammed, Kei Long Cheung, Bjorn Winkens, Nanne de Vries, Hein de Vries

**Affiliations:** 1 Faculty of Health, Medicine and Life Sciences, Care and Public Health Research Institute, Maastricht, Netherlands; 2 Department of Clinical Sciences, College of Health and Life Sciences, Brunel University, London, United Kingdom

**Keywords:** adolescents, I-Change Model, smoking initiation, smoking predictors, Saudi Arabia

## Abstract

**INTRODUCTION:**

Knowing country-specific predictors of smoking behaviour for adolescents is crucial for successful smoking prevention programs. This study aims to assess demographic and socio-cognitive variables related to smoking initiation among Saudi male adolescents.

**METHODS:**

Longitudinal data were collected at T1 (baseline) and at T2 (followup at 6 months) using a self-administered questionnaire. We assessed smoking behaviour and related demographic variables and socio-cognitive variables. Chi-squared tests and independent-samples t-tests were used to identify differences in baseline characteristics between smokers and non-smokers at T1. Furthermore, non-smokers at T1 were included in logistic regression analyses to examine the predictors of smoking initiation between T1 and T2.

**RESULTS:**

At T1, the non-smokers who were included in further analysis were 523 (84.9%) of whom 48 (9.2%) had initiated smoking at T2. They differed significantly from non-initiators, including having a more positive attitude towards smoking, reporting more social norms, modelling and pressure to smoke, having a lower self-efficacy to refrain from smoking and higher intention to smoke in the future (all p<0.001). The regression analysis revealed that: adolescents with disrupted-families, being of low academic achievement, with relatively high monthly-income families, having more smoking-peers, high-perceived pressure to smoke from parents (p=0.002) and teachers (p=0.001), have smoking supportive-norms of parents and having high intention to smoke in the future (p<0.001) were at higher risk of being smokers.

**CONCLUSIONS:**

Findings suggest that health-promoting programs should address strengthening of self-efficacy and enhancing refusal skills against modelling of peers, pressure and norms of parents.

## INTRODUCTION

Smoking remains one of the major public health problems. It is associated with different types of morbidities, including cancers, heart and coronary diseases, and lung disease; almost all systems of the body are negatively affected by smoking^1,2^. Smoking is the number one preventable cause of death^3^.

Smoking experimentation and initiation mostly occurs during adolescence^4^; approximately 40% of smokers start by this age^5^ and it is estimated that 88% of adults who smoke daily started smoking by the age of 18 years^6^. Early smoking initiation is associated with difficulty in quitting, being regular smokers as adults^7^ and with susceptibility to addiction^8^.

The Integrated Change Model (I-Change Model)^9,10^ integrates several cognitive models to understand and change health behavior. It originated from the Attitude-Social influence-Self efficacy (ASE) model, which is based on the Theory of Reasoned Action^11^. The model incorporates insights from the Transtheoretical Model^12^, the Theory of Planned Behavior^13^, Social Cognitive Theory^14^, the Precaution Adoption Model^15^, and goal setting theories^16^. The model assumes that intention is the most proximal predictor of behavior, which in turn is influenced by a person’s attitude comprising cognitive and emotional advantages and disadvantages, social influence beliefs (i.e. norms about smoking, smoking behavior by others, and social pressure), and self-efficacy. Additionally, the most recent version acknowledges pre-motivational and post-motivational determinants^10^.

Investigation of smoking behavior predictors among adolescents is an essential step to develop and design a successful smoking prevention program. The social influence approach to study smoking behavior was developed for the first time by Evans in 1976 when with colleagues addressed the impact of social pressure to smoke from parents, media, and peers^17^. In 1994 the report of the Surgeon General of the United States Department of Health and Human Services clearly showed that smoking initiation was associated with psychological and social factors^18^. Using longitudinal data, De Vries et al.^19^ found that the social influence constructs (social norms, perceived smoking behavior, and direct pressure), in additon to self-efficacy and intention were significant predictors of adolescent smoking behavior. In a review with 53 longitudinal studies, published between January 1984 and August 2015, ninety-eight potential predictors were identified, including increased age/grade, poor academic performance, lower socioeconomic status, intention to smoke in the future, smoking family members, smoking friends, and exposure to smoking promoting films and tobacco promotion efforts, against which high self-efficacy was found to be protective^20^.

In Saudi Arabia, several studies investigated smoking predictors among adolescents. Results were similar to those found in international studies. Al-Zalabani et al.^21^ in their cross-sectional study found that having smoking friends, parental smoking, exposure to cigarette advertisements in mass media, and higher pocket money were risk factors for smoking initiation. Al-Makadma et al.^22^ in their survey documented that paternal smoking was the most significant factor to explain smoking onset; their findings were supported by Alsubaie^23^ who also found age, studying in private schools, having friends who smoke, perceived poor health and dissatisfaction with life as predictors of smoking onset.

The aim of this paper is to investigate the predictors of smoking intiation among Saudi male adolescents to guide the development of smoking prevention programs for this target group.

## METHODS

### Sampling

As part of the development of a smoking prevention program targeting school going adolescents in Taif, Saudi Arabia, secondary schools were approached to participate in a two-armed cluster randomized controlled trial. Nine schools were randomly selected to represent the control group. To select participants, students were given the chance to pick one of two papers in which either ‘Included’ or ‘Excluded’ were written. Out of the 707 included, twenty-four students did not fill in the questionnaire, resulting in 683 (96.6%) participants. In the current study, we analyze the data of the control group only for whom baseline (T1) and at 6 months (T2) longitudinal data were obtained. Only boys were included in the study, since the educational system in Saudi Arabia is gender specific and smoking is officially not considered a problem for girls^24^.

### Ethical approval

The data were collected with the approval of the General Directorate of Education, school health program and school masters. Participants were given the right not to participate or stop at any time, as was explained prior to filling in the questionnaire.

### Questionnaire

The questionnaire used was a modified version of the European Smoking Prevention Framework Approach (ESFA) based on the I-Change Model^10,25^. Translation to Arabic and back-translation was done by a public-health expert, with some modifications to fit with Saudi norms and culture. The questionnaire was pretested in a focus group discussion for participants from the same selected schools and accordingly some adaptations were made. The questionnaire assessed demographics, attitudes, social influences, self-efficacy, intention not to smoke in the future and smoking behavior.

Demographic factors included were: age (months), school area (coded as rural=1, urban=2), school type (public=1, private=2), family monthly income (US$) (<800 = 1; ≥800 and <1600 = 2; ≥1600 and <2400 = 3; ≥2400 = 4), daily pocket money (US$) (<2 = 1; ≥2 = 2), academic performance for the last year final exam (higher = 1, middle = 2 or lower third of the class = 3), and family structure (stable family, i.e. lives as one family with father and mother = 1, disrupted family, i.e. lives with father and mother but the father has another wife, parents are divorced, or one or both parents are dead = 0).

Attitude was assessed using a 7-point Likert scale with 9 items: 1) very pleasant/very unpleasant, 2) very desirable/very undesirable, 3) makes me feel very relaxed/makes me feel very stressed, 4) very much more confident to be part of the crowd/ very much less, 5) very much friendly/very much unfriendly, 6) very much sociable/very much unsociable, 7) tastes really very nice/tastes really very horrible, 8) friends pay much more attention if I smoke/pay much less attention, and 9) much more easier to start talking with others/much more difficult; coded as (+3, -3) (Cronbach’s α for T1=0.78, α for T2=0.79).

Social influence beliefs were measured using three constructs: social modeling, social norms, and perceived social pressure^19^. Social modeling was assessed by asking respondents to indicate the smoking behavior of important people in their environment (father, mother, brother, sister, best friend, friends, teachers and classmates) (does not smoke/the majority does not smoke = -1, do not know/not present = 0, smokes/the majority smokes =1) (αT1=0.67, αT2=0.69). Social norms were measured by asking whether respondents believed that these people felt that they should smoke or not (definitely yes = 3, definitely no = -3) (αT1=0.97, αT2=0.98). To measure perceived social pressure to smoke the respondents were asked whether they countered pressure to smoke from the same eight people (never = 0, few times = 1, sometimes = 2, often = 3 and very often = 4) (αT1=0.86, αT2=0.88).

Self-efficacy was assessed using a 7-point Likert scale by asking the adolescents to indicate how difficult they found it not to smoke in a variety of situations (very difficult not smoke = +3; very easy not to smoke = -3). The situations measured were: with another who smokes, with friends who smoke, when offered a cigarette by someone, or by a friend, on the way home from school, while watching TV, doing homework, going out with friends, stressed, upset, depressed, nervous, worried, and when shopping (αT1=0.98, αT2=0.98).

Intention not to smoke was asked by four items on a 7-point scale to assess whether adolescents had the intention to smoke in the next 6 months, the next year, the next five years and in the future (definitely yes = 3; definitely no = -3) (αT1=0.98, αT2=0.98).

Smoking status was defined when the adolescent indicated that he had smoked at least once a week or daily, and when he reported that he smoked 100 cigarettes or more, unless he reported that he had quit. An adolescent was coded as a non-smoker when he had quit smoking, smoked monthly/occasionally, or experimented with smoking. An algorithm with four questions was used to validate the self-reported smoking status (number of cigarettes smoked last day, last week, last month and lifetime number of smoked cigarettes^10,25^; any inconsistency in response was resolved by recoding to the most unfavorable response^25^.

### Statistical analysis

Chi-squared tests and t-tests were used to assess the baseline differences in demographic and sociocognitive factors between smokers and non-smokers at T1, and between smoking initiators and not smoking initiators, where smoking initiation is assessed between T1 and T2. Logistic regression analyses were used to identify independent risk factors of smoking initiation between T1 and T2. As in the order prescribed by the I-Change Model, three separate models were built using a forward stepwise selection model, as the number of potential risk factors was too large to be included all at once in a model. The following variables were considered to be included in the models: only demographics in Model 1, demographics plus motivational constructs (attitude, social influences [modeling, pressure and norms] and self-efficacy) in Model 2, and demographics, motivational constructs, and intention in Model 3. The variables, which were significantly related to the outcome in Model 1, were also considered to be included in Model 3, but did not appear in the final Model 3 as they were no longer significant. In all analyses, the dependent variable was smoking status at T2, where only non-smokers at T1 were included to address smoking initiation between wave 1 (T1) and 2 (T2). As sensitivity analysis, the three final logistic regression models were repeated with the general estimated equation (GEE) analysis, accounting for the nesting of adolescents within schools (exchangeable structure).

Assumptions were checked using variance inflation factors (VIF>10 indicates a (multi) collinearity problem), Cook’s distances (>1 indicates an influential outlier problem), and tests on quadratic terms (if the centered quadratic term is significant, the linearity assumption is violated).

All data analyses were performed using IBM SPSS Statistics for Windows (version 24.0, Armonk, NY: IBM Corp). Two-sided p-values ≤0.05 were considered statistically significant.

## RESULTS

Of the 707 adolescents, 683 (96.6%) filled in the questionnaire at both time intervals; 67 of these were excluded from the analysis either for not fully completing the questionnaire or for missing important demographic and cognitive variables, resulting in 616 out of 707 (87.1%) participants included in the analysis.

The mean age of the included adolescents (n=616) at T1 was 13.5 years (SD=0.49). The vast majority 565 (91.7%) lived in urban areas and most studied at public schools 540 (87.7%). The distribution of the respondents based on family structure was: 520 (84.4%) lived in a stable family with father and mother, while 96 (15.6%) lived in disrupted families, including 46 (7.5%) with father and mother but the father had another wife, 32 (5.2%) had divorced parents, and of the remaining 18 (2.9%) one or both parents had passed away. As for daily pocket money, 48.7% got less than US$2. The families of 217 (35.2%) participants earned more than US$2400 per month, 146 (23.7%) between US$1600 and US$2400, 104 (16.9%) between US$800 and <US$1600, and 149 (24.2%) earned <US$800. At T1, there were 523 (84.9%) non-smokers and 93 (15.1%) smokers. Smokers at T1 were more often from a disrupted family, had more daily pocket money, and belonged less often to the higher third of the class than non-smokers (all p<0.001) (Table 1).

**Table 1:** Sociodemographic characteristics of the respondents distributed by smoking status at T1, for 616 adolescents, Saudi Arabia

*Items*	*Categories*	*Non-smokers N= 523 (84.9%) n (%)*	*Smokers N=93 (15.1%) n (%)*	*p*
Family structure	Stable family	445 (85.6)	75 (14.4)	<0.001
	Disrupted family	78 (81.3)	18 (18.7)	
Family monthly income (US$)	<800	123 (82.6)	26 (17.4)	0.06
	≥800 and <1600	94 (90.4)	10 (9.6)	
	≥1600 and <2400	130 (89.0)	16 (11.0)	
	≥2400	176 (81.1)	41 (18.9)	
Daily pocket money (US$)	<2	275 (91.7)	25 (8.3)	<0.001
	≥2	248 (78.5)	68 (21.5)	
Academic performance	Among the higher third of the class	286 (94.0)	18 (6.0)	<0.001
	Among the middle third of the class	150 (80.2)	37 (19.8)	
	Among the lower third of the class	87 (69.6)	38 (30.4)	
School area	Urban	483 (85.5)	82 (14.5)	0.18
	Rural	40 (78.4)	11 (21.6)	
School type	Public	462 (85.6)	78 (14.4)	0.23
	Private	61 (80.3)	15 (19.7)	

To identify the predictors of smoking onset, only the non-smokers at baseline 523 (84.9%) were included in further analyses. Of these non-smokers at T1, 475 (90.8%) had not initiated smoking at T2, whereas 48 (9.2%) initiated smoking between T1 and T2. Adolescents who did not initiate smoking scored on average significantly higher on self-efficacy, and lower on attitude towards smoking and intention to smoke than smoking initiators (all p<0.001). The analysis of social influence items showed that smoking initiators scored significantly higher than non-initiators on all items assessed (all p<0.001), except for the social pressure from the mother (p=0.32) and sister (p=0.68), and modeling of mother (p=0.35) and sister (p=0.09). Analysis of the perceived social influence of parents and peers led to significant differences between the two groups for the three constructs assessed (Table 2).

**Table 2:** T-test for the cognitive factors at T1 and smoking initiation between T1 and T2, for 523 adolescents, Saudi Arabia

*Items*	*Nonsmokers N=475*		*Smokers N=48*		*p*
	*M*	*SD*	*M*	*SD*	
Attitude^a^	-1.31	1.04	0.04	1.96	<0.001
Self-efficacy^e^	1.20	0.66	0.47	0.70	<0.001
Social pressure − all^a^	0.28	0.55	1.57	0.70	<0.001
Mother^b^	0.00	0.00	0.08	0.58	0.32
Father^b^	0.04	0.37	0.29	0.83	<0.001
Brother^b^	0.15	0.67	1.60	1.46	<0.001
Sister^b^	0.05	0.38	0.08	0.58	0.57
Friends^b^	0.54	1.09	3.25	1.30	<0.001
Best friend^b^	0.55	1.30	3.27	1.32	<0.001
Classmates^b^	0.60	1.11	2.58	1.64	<0.001
Teacher^b^	0.27	0.91	1.40	1.30	<0.001
Parents	0.02	0.18	0.19	0.64	0.08
Peers	0.38	0.73	2.16	0.90	<0.001
Social Norms − all^c^	-1.81	1.21	1.20	1.18	<0.001
Mother^c^	-2.20	1.27	0.83	1.74	<0.001
Father^c^	-2.18	1.25	1.27	1.05	<0.001
Brother^c^	-2.00	1.51	1.46	1.29	<0.001
Sister^c^	-1.97	1.27	0.79	1.82	<0.001
Friends^c^	-1.47	1.64	1.06	1.66	<0.001
Best friend^c^	-1.82	1.58	1.06	1.69	<0.001
Classmates^c^	-1.33	1.63	1.65	0.76	<0.001
Teachers^c^	-1.54	1.64	1.44	0.92	<0.001
Parents	-2.19	1.20	1.05	1.25	<0.001
Peers	-1.71	1.21	1.20	1.29	<0.001
Social Modeling − all^d^	-0.69	0.41	0.22	0.29	<0.001
Mother^d^	-0.97	0.24	-1.00	0.00	0.35
Father^d^	-0.59	0.78	0.63	0.76	<0.001
Brother^d^	-0.71	0.68	0.79	0.62	<0.001
Sister^d^	-0.92	0.35	-1.00	0.00	0.09
Friends^d^	-0.63	0.70	0.67	0.60	<0.001
Best friend^d^	-0.53	0.78	0.90	0.42	<0.001
Classmates^d^	-0.68	0.66	0.38	0.91	<0.001
Teachers^d^	-0.47	0.80	0.44	0.87	<0.001
Parents	-0.77	0.46	-0.19	0.38	<0.001
Peers	-0.70	0.45	0.35	0.39	<0.001
Intention^f^	-1.90	1.45	1.56	1.34	<0.001

a Very desirable=3, Very undesirable=-3. Very pleasant=3, Very unpleasant=-3, etc.

b Never=0, Very often=4.

c Definitely should smoke=3, Definitely should not smoke=-3.

d No=-1, I don’t know=0, Yes=1.

e Very hard not to smoke=-3, Very easy not to smoke=3.

f I am sure I will smoke=3, I am sure I won’t smoke=-3.

In order to identify the predictors of smoking onset, we ran three logistic regression models, where all assumptions were met. If we only consider demographics (Model 1), adolescents with low academic performance, who were members of a disrupted family and had a relatively high family monthly income were more vulnerable to initiate smoking than others. From Model 2, including demographics and motivational constructs, adolescents with more smoking peers (brothers, sisters, friends, best friend, and classmates) were at higher risk of being smokers too. Also, those with high perceived social norms of parents and with pressure to smoke from parents (mostly the father) or teachers were more likely to smoke. Adding intention to demographics and motivational constructs (Model 3) suppressed the effect of peers modeling, and teachers’ pressure, while perceived pressure and norms of parents remained significant in the final model (Table 3).

**Table 3:** Results of logistic regression analysis for factors associated with smoking initiation between T1 and T2, for 48 smoking initiators, Saudi Arabia

	*Items*	*OR*	*95% CI*	*p*
Model 1	Family structure	0.07	0.03−0.17	<0.001
	Academic performance (Ref. group: lower third of the class)			<0.001
	Among the highest third of the class	0.03	0.01−0.13	<0.001
	Among the middle third of the class	0.22	0.09−0.56	0.001
	Family monthly income (US$) (Ref. group: ≥2400)			0.05
	<800	0.97	0.25−3.83	0.97
	≥800 and <1600	1.32	0.37−4.74	0.68
	≥1600 and <2400	3.99	1.35−11.82	0.01
Model 2	Family structure	0.10	0.03−0.35	<0.001
	Academic performance (Ref. group: lower third of the class)			0.05
	Among the highest third of the class	0.13	0.02−0.85	0.03
	Among the middle third of the class	0.36	0.11−1.17	0.09
	Social model peers^a^	7.77	2.38−25.37	0.001
	Social norms parents^b^	2.39	1.60−3.58	<0.001
	Social pressure parents^c^	4.25	1.68−10.75	0.002
	Social pressure teachers^c^	0.47	0.30−0.72	0.001
Model 3	Social norms parents^b^	2.80	1.92−4.09	<0.001
	Social pressure parents^c^	4.52	1.69−12.11	0.003
	Intention^d^	3.00	2.07−4.35	<0.001

a No=-1, Don’t know or don’t have this person=0, Yes=1.

b Definitely should smoke=3, Definitely should not smoke=-3.

c Never=0, Very often=4.

d I am sure I will smoke=3, I am sure I won’t smoke=-3.

The sensitivity analyses with GEE, accounting for nesting of adolescents within schools, showed similar results for all three models (intra-class correlation ICC<0.001).

## DISCUSSION

Our findings about smoking behavior and academic performance support the findings of other studies and reviews in which smoking was more prevalent in adolescents with lower academic performance^20,26^. In line with other studies^25,27^, smoking prevalence was higher among respondents from disrupted families. Smokers and non-smokers varied significantly in received daily pocket money, while family monthly income revealed the trend that smoking prevalence was higher among respondents of higher socioeconomic status, in contrast to findings of similar studies in other countries that documented that uptake is higher among those with low socioeconomic status^28,29^. Yet, our result is consistent with the findings from local studies^23,30^. This may indicate that the smoking epidemic is at an early stage in Saudi Arabia and, as according to Lopez et al.^31^, smoking is initially highest among people in higher socioeconomic strata, an alarming fact that the tobacco control in Saudi Arabia should consider.

Smokers and non-smokers differed significantly for all assessed social influence constructs, except for perceived modeling and pressure to smoke from mothers and sisters, which is likely to be due to the nature of the Saudi community where males and females are raised separately with boys being more attached to fathers and girls to mothers. Smokers perceived higher pressure to smoke, more often had smoking parents (mostly fathers), peers and teachers, experienced smoking supportive norms, had positive attitudes towards smoking, less self-efficacy to refrain from smoking and a high intention to smoke in the future. Our findings are in line with several international studies^11,17,19,32^ showing the importance of the social influence approach in understanding adolescent smoking behavior.

To examine the predictors for smoking initiation we ran logistic regression analyses. Model 1 with only sociodemographic factors revealed that academic performance and family structure were significant predictors. Model 2, which added the motivational factors^33^, revealed a significant impact of perceived parental norms and pressure to smoke and modeling of peers; findings also reported by other local^21-23^ and international studies^18,34^. Model 3 added intention to the previous factors and revealed that intention to smoke in the future was a highly significant predictor of smoking initiation; findings also supported by other studies^35^. Parental norms and pressure remained significant in this model; this finding was also found in another study that reported that social influence can act directly on behavior^32^.

### Limitations

Our study had some limitations. First, only boys were included, because smoking among females was not considered a public health problem in Saudi Arabia; hence, we could not get approval from girls’ schools. Secondly, six months is considered a relatively short period for behavioral change. Thirdly, we could not biologically validate self-reports of smoking behavior. However, it is clearly documented that there is high correlation between biochemical assessment of adolescent smoking behavior and self-reports, if confidentiality is preserved and anonymity is assured^36^, an approach that we also followed.

## CONCLUSIONS

Finding of this study can help the school health program and tobacco control program in Saudi Arabia in shaping smoking prevention programs for adolescents. Coping with pressure to smoke, refusal skills development and enhancing self-efficacy are essential elements to be considered for better outcomes from anti-smoking interventions.
